# An Observational Study to Find the Association of Viral Load, NS1 Antigen, IgG Antibody, and Other Laboratory Parameters With the Outcome of Dengue Patients in Eastern India

**DOI:** 10.7759/cureus.63516

**Published:** 2024-06-30

**Authors:** Soma Sarkar, Anindya Ghosh, Soumi Nag, Shantanab Das, Dipankar Sarkar

**Affiliations:** 1 Microbiology, Infectious Diseases and Beleghata General Hospital, Kolkata, IND; 2 Microbiology, Panihati State General Hospital, Kolkata, IND; 3 Human Genetics, Indian Statistical Institute, Kolkata, IND; 4 Critical Care Medicine, Manipal Hospitals Salt Lake, Kolkata, IND

**Keywords:** liver enzymes, secondary infection, ferritin, platelet, outcome of dengue, ns1 antigen, viral load, dengue

## Abstract

Background

Dengue, the mosquito-borne febrile disease caused by the dengue virus, has become one of the major concerns of public health. It may present with only fever, or there may be a hemorrhagic manifestation or septic shock. As there is no specific treatment for dengue, early detection of the disease, assessment of progression, and prediction of outcome by studying the laboratory markers will help guide the management of cases and lower morbidity and mortality.

Methodology

This clinico-observational study was conducted at the Department of Microbiology in a tertiary care hospital in Kolkata, India, from February 2020 to August 2022 to determine the outcome of dengue patients in correlation with viral load, NS1 antigen, IgM and IgG antibodies, ferritin level, platelet count, and other laboratory parameters.

Results

Out of 316 samples from fever patients, 103 (32.5%) were NS1 antigen reactive. We followed up the dengue patients (n = 103) for 15 days and divided them into three groups according to their duration of symptoms (group A suffered for ≤5 days, group B for 5 to 10 days, and group C for >10 days) and per the WHO classification of disease severity, namely dengue without warning signs (DOS), dengue with warning signs (DWS), and severe dengue (SD). Based on severity, 65 (63.1%) patients had DOS, whereas 31 (30.09%) patients had DWS, and seven (6.79%) patients had SD. Secondary infection was present in 83.33% of patients in group C, 71% of DWS cases, and 57% of SD cases, which positively correlates with liver enzymes, viral load (mean value 102195 in secondary infection vs. 1195 copies/10 µl in primary infection), and negatively correlates with platelet counts (mean value 60,213 in secondary infection vs. 1,25,516 in primary infection). Patients in group C had higher liver enzymes, a lower platelet count, and a higher initial viral load than groups A and B. Similarly, SD cases had a higher ferritin level (9215 ug/l), a lower platelet count (mean value 23,250), and a higher initial viral load (mean value 2,74,257 copies/10 µl). An increase in hematocrit value considering the peak value and its baseline value is an important marker for disease severity rather than its absolute value.

Conclusion

Poor outcome of dengue infection, i.e., an increase in the duration of symptoms and disease severity depends on concurrent associations between high serum ferritin, increased hematocrit level, thrombocytopenia, secondary infection, increasing liver enzymes, and increased initial viral load.

## Introduction

Dengue, the mosquito-borne febrile disease, is one of the most prevalent arboviral infections affecting humans throughout the world. Over the years, dengue has become one of the major concerns of public health. The number of dengue cases has increased 10-fold over the last two decades (from 505,430 cases in 2000 to over 2.4 million in 2010, and 5.2 million in 2019), and 75% of the global population exposed to dengue is in the Asia-Pacific region [1]. After a slight decline in cases between the years 2020 and 2022 due to the COVID-19 pandemic and lower reporting rate, in 2023, an upsurge in dengue cases has been observed globally [2]. The disease is caused by a flavivirus, which has five serotypes: DENV1, DENV2, DENV3, DENV4, and DENV5. The fifth one, DENV-5, was isolated in October 2013 from Bangkok [3] and follows a sylvatic cycle.

*Aedes aegypti* is the main vector in the transmission of dengue, followed by *Aedes albopictus* and *Aedes polynesiensis* according to the geographic distributions [4,5]. After a three-to-seven-day incubation period, nonspecific symptoms like fever, headache, retro-orbital pain, myalgias, and petechial rashes occur in infected individuals that last for three to seven days.

The first phase of the clinical course is the febrile phase, which lasts for one to three days. Mild hemorrhagic manifestations such as petechiae and mucosal membrane bleeding (e.g., nose and gums) may be seen. In the critical phase, the temperature drops at first and then may rise; most of the complications occur in this phase. The recovery phase may be uneventful, or complications may occur. Risk factors such as older age, diabetes, heart disease, obesity, hypertension, hemolytic conditions, prior dengue infection with other serotypes, and pregnancy may complicate the disease course.

The main pathologies behind dengue hemorrhagic fever (DHF) and dengue shock syndrome (DSS) are increased capillary permeability and thrombocytopenia. Depending on symptoms, the WHO in 2009 classified DENV-infected patients into dengue without warning signs (DOS), dengue with warning signs (DWS), and severe dengue (SD) [6]. For febrile patients with suspected dengue within the first five days, detection of dengue virus RNA by reverse transcriptase real-time PCR (rRT-PCR) or detection of dengue NS1 antigen by immunoassay is the preferred method of laboratory diagnosis [7]. The serum ferritin, an acute-phase reactant, measured after the third day of fever may predict the chances of SD [8]. The high ferritin levels are associated with the pro-inflammatory cytokine release [9]. Later in the illness (≥5 days after onset of fever), IgM against dengue virus by membrane attack complex by enzyme-linked immunosorbent assay (MAC-ELISA) should be done.

Infection provides lifelong immunity to the same serotype, but cross-protection to other serotypes is of short duration. So secondary infection by different serotypes leads to antibody-dependent enhancement (ADE), which can cause a higher viral load and more mononuclear cell activation, which leads to cytokine storm [10]. As there is no specific treatment for dengue, early detection of the disease, assessment of progression, and prediction of outcome by studying the laboratory markers will help guide the management of cases and lower morbidity and mortality.

## Materials and methods

After getting approval from the Institutional Ethics Committee of Nilratan Sircar Medical College and Hospital, Kolkata, India (approval no. NMC/697), this clinico-observational study was conducted at the Department of Microbiology of the tertiary care hospital from February 2020 to August 2022 to determine the outcome of dengue patients in correlation with viral load, NS1 antigen detection, IgM and IgG detection, ferritin level, platelet count, and other laboratory parameters.

Patient recruitment and sample collection

Around 10 ml of venous blood was collected under aseptic conditions from febrile patients of all age groups and both sexes attending the outpatient department (OPD) as well as those admitted in different wards with clinical suspicion of dengue (with a history of fever <5 days). Exclusion criteria were fever for >5 days, presence of any other source of infection (e.g., malaria, coronavirus disease, otitis media, pneumonia, meningitis), or any other chronic illness. Only dengue NS1 antigen-positive patients were followed up for further study.

Detection of dengue NS1 antigen

Whole blood was allowed to clot, and the serum was separated after centrifugation. The dengue NS1 antigen ELISA was done by using the dengue NS1 ELISA kit (Bhat Bio-Tech India Pvt. Ltd., Bangalore, KA, IND) and the DENV Detect (InBios International Inc., Seattle, WA, USA) according to the manufacturer's instructions.

Serum ferritin level

On the third or fourth day of fever in dengue NS1-positive cases, the serum ferritin level was checked using the electrochemiluminescence immunoassay analyzer (ECLIA) (Cobas 6000, Roche Diagnostics, Indianapolis, IN, USA). The serum ferritin level is considered high if it is more than 500 ng/ml [11].

The WBC count, platelet count, hematocrit, and liver function tests

A complete blood count (CBC) was done using the Sysmex KX-21 hematology analyzer (Bioprom Medical Supplies, Thessaloniki, Thermi, GRC), and liver function tests to measure the liver enzymes serum glutamic oxaloacetic transaminase (SGOT), serum glutamic pyruvic transaminase (SGPT), and alkaline phosphatase (ALP) were performed by the Konelab autoanalyzer (Thermo Fisher Scientific, Waltham, MA, USA). The level during recovery at which patients were hemodynamically stable was taken as baseline hematocrit. The increase in hematocrit percentage was calculated as: [(peak hematocrit - baseline hematocrit) / baseline hematocrit] x 100. A value of 20% or more was considered significant hemoconcentration.

Dengue viral load estimation

For viral load estimation, RNA extraction from serum was done using the QIAamp Viral RNA Mini Kit (Qiagen, Hilden, DEU) using the manufacturer's protocol. The HELINI Dengue RT-PCR Kit (Helini Biomolecules, Chennai, TN, IND) was used to detect viral load for each sample using the manufacturer's instructions.

Detection of dengue IgM and IgG antibody

To detect dengue IgM and IgG antibodies by ELISA, the DENV Detect™ IgM Capture ELISA Kit and DENV DetectTM IgG kit (InBIOS International Inc.) were used. The ELISA was done as per the manufacturer’s protocol. Secondary infection was considered in those cases that had a primary infection previously and were later infected by any of the other three serotypes of the dengue virus. Secondary dengue infection was defined as samples that had IgG only or IgG/IgM ≥ 1.2 [12].

Statistical analysis

All calculations were performed using SPSS Statistics version 26.0 (IBM Corp., Armonk, NY, USA). Laboratory data were presented as descriptive statistics, including number, percentage, and mean. The chi-square test was used to compare categorical variables. A p-value <0.05 was considered significant.

## Results

Detection of dengue NS1 antigen

**Figure 1:**
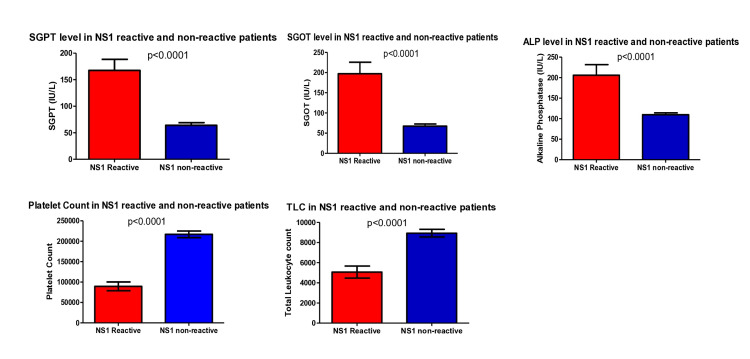
Liver enzymes and total leukocyte count in NS1 reactive patients compared to the NS1 non-reactive cases SGPT: Serum glutamic pyruvic transaminase, SGOT: Serum glutamic oxaloacetic transaminase, ALP: Alkaline phosphatase, TLC: Total leukocyte count

Out of 316 samples from fever patients, 103 (32.5%) were NS1 antigen reactive. Amongst the NS1 antigen-reactive patients, the majority, i.e., 77 (74.75%), were aged between 14 and 50 years, 65 (63.10%) were male, and 38 (36.90%) were female. There was a significant increase in liver enzymes and a decrease in platelets as well as total leukocyte count in NS1 reactive patients compared to the NS1 non-reactive cases (Figure 1).

**Figure 2:**
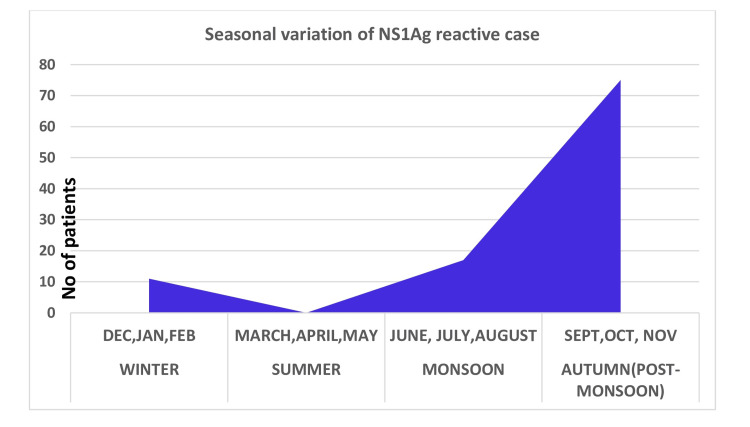
Distribution of NS1 antigen reactive patients as per season Ag: Antigen

The highest burden of disease was seen during the post-monsoon period (72.8%, n = 75) spanning from September to November, followed by the monsoon (16.5%, n = 17) and winter (10.6%, n = 11). There was no NS1 reactive sample in the summer (Figure 2).

All NS1-positive patients presented with fever, chills (100%), and arthralgia (100%). The majority of the patients, i.e., 82 (79.61%) presented with retro-orbital pain; 78 (75.72%) had abdominal pain; 64 (62%) had headache and vomiting; 59 (57.28%) had a rash; and three (2.91%) had seizures. Various laboratory parameters like dengue IgG status, leukocyte count, platelet count, hematocrit, liver enzymes, serum ferritin, and viral load were monitored. As there was no mortality in this study period, we analyzed the outcome based on the duration of illness (time taken to become free of signs and symptoms) and severity of the disease. So, we divided the dengue patients (n = 103) into three groups according to their duration of illness: those who suffered ≤ 5 days in group A (n = 34), between five and 10 days were in group B (n = 51), and >10 days were in group C (n = 18). Based on the severity of the disease we found 65 (63.1%) patients had DOS, whereas 31 (30.09%) patients were found to have DWS and seven (6.79%) had SD.

Correlation of primary and secondary dengue infection with duration of disease

**Figure 3:**
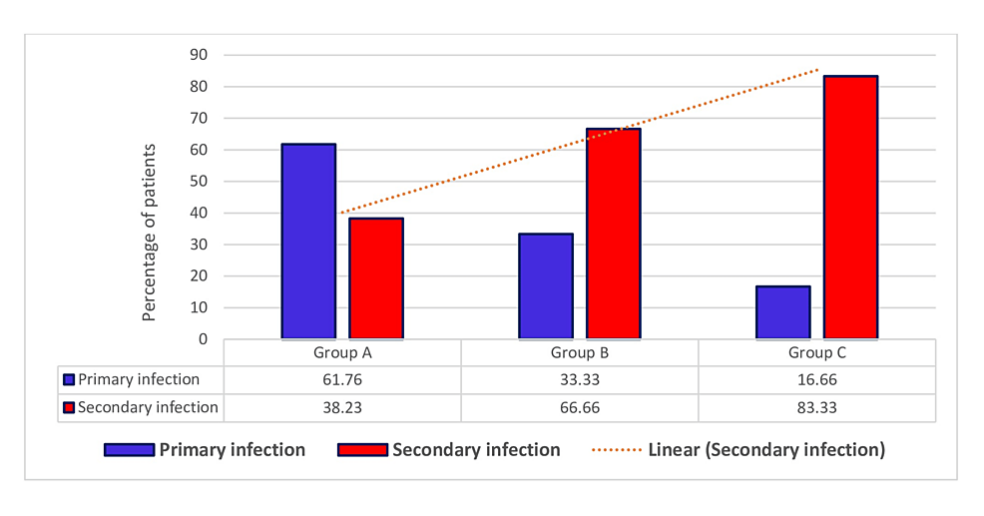
Correlation of primary and secondary dengue infection with disease duration

Among 103 NS1 reactive cases based on IgG and IgM ratio, 58 cases (56.31%) had secondary infection and 45 (43.69%) had only primary infection. The groupwise rate of secondary infection is shown in Figure 3. Around 83.33% of group C patients (n = 18) had secondary infection and had more days of illness.

Correlation of primary and secondary dengue infection with disease severity

**Figure 4:**
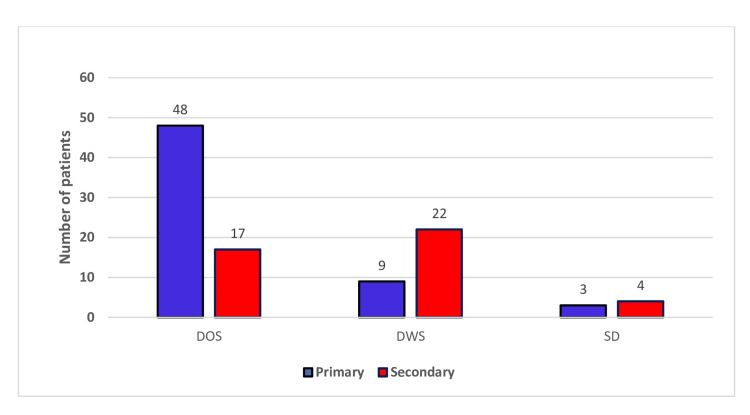
Correlation of primary and secondary dengue infection with disease severity DOS: Dengue without warning signs, DWS: Dengue with warning signs, SD: Severe dengue

Among the 103 dengue patients, 71% of DWS cases and 57% of SD cases were found to be associated with secondary dengue infection (Figure 4). 

Association between dengue IgG status with liver enzymes and platelet count in NS1 reactive patients

**Figure 5:**
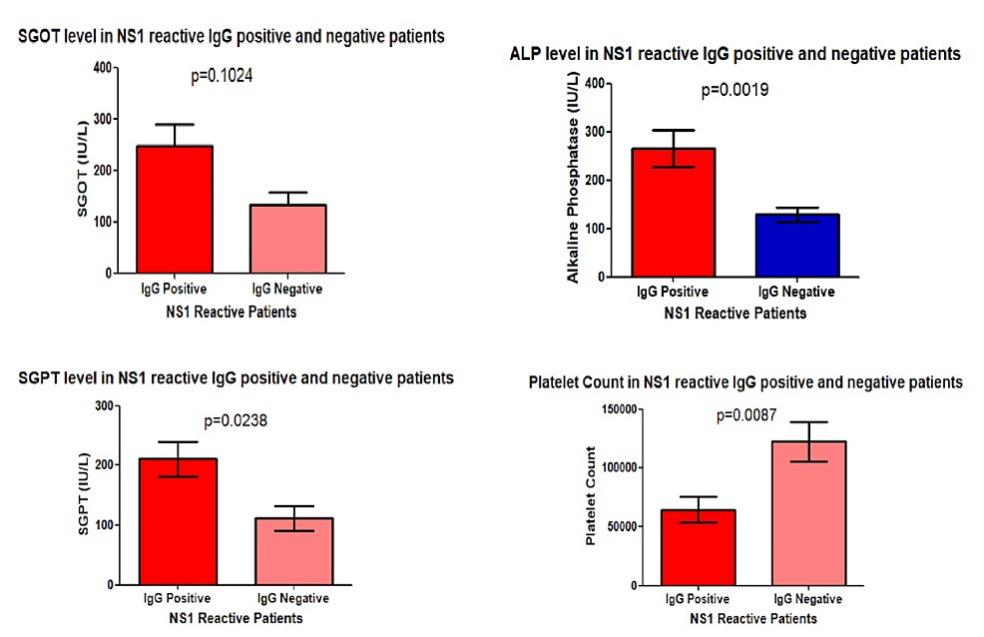
Association between dengue IgG status with liver enzymes and platelet count in NS1 reactive patients SGPT: Serum glutamic pyruvic transaminase, SGOT: Serum glutamic oxaloacetic transaminase, ALP: Alkaline phosphatase

Among NS1 reactive samples, those who were dengue IgG reactive positively correlated with liver enzymes, i.e., they had higher levels of liver enzymes than IgG non-reactive patients but had a negative correlation with platelet counts (Figure 5).

Correlation of liver enzymes, platelet counts, and viral load with duration of illness

**Figure 6:**
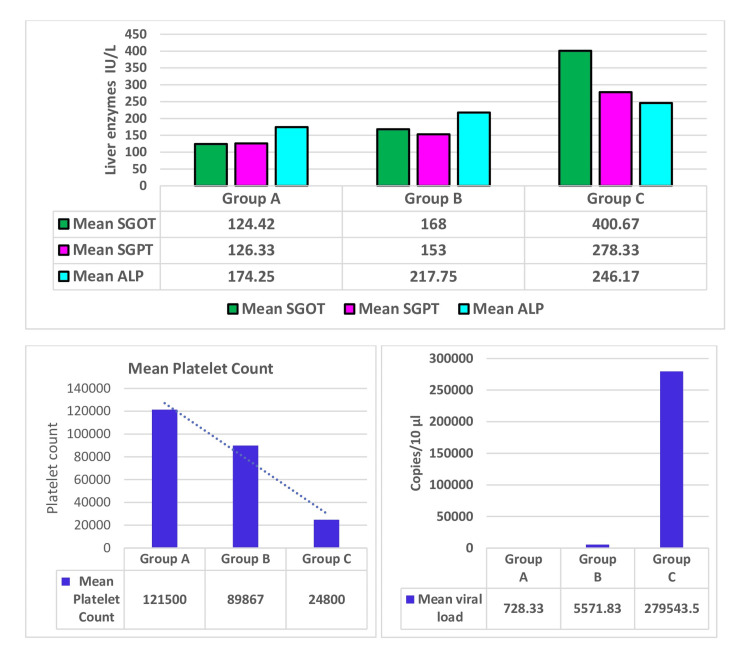
Correlation of liver enzymes, platelet counts, and viral load with duration of illness SGPT: Serum glutamic pyruvic transaminase, SGOT: Serum glutamic oxaloacetic transaminase, ALP: Alkaline phosphatase

The mean SGOT, SGPT, and ALP in group A were 124.42, 126.33, and 174.25 IU/L, respectively. In group B, it was 168.0, 153.50, and 217.75 IU/L, respectively. In group C, it was 400.67, 278.33, and 246.17 IU/L, respectively. The liver enzymes (mean SGOT, SGPT, and ALP) were higher in group C than in group B and group A. It was also found that liver enzymes (mean SGOT, SGPT, and ALP) increase with the duration of illness and tend to elevate more in secondary dengue infection than in primary infection. Again, the mean platelet count in groups A and B was 121500 and 89866.67/cumm, respectively, while in the case of group C, the mean platelet count was found to be very low, i.e., 24800/cu mm. Platelet count decreases more with patients having more days of illness, but in total leukocyte count, there was no such correlation with secondary infection or disease severity. Considering the mean viral load, it was 728.33 copies /10 µl in group A, 5571.83 copies /10 µl in group B, and 279543.50 copies /10 µl in group C (Figure 6).

Correlation of dengue viral load, ferritin level, hematocrit, and platelet count with disease severity

**Figure 7:**
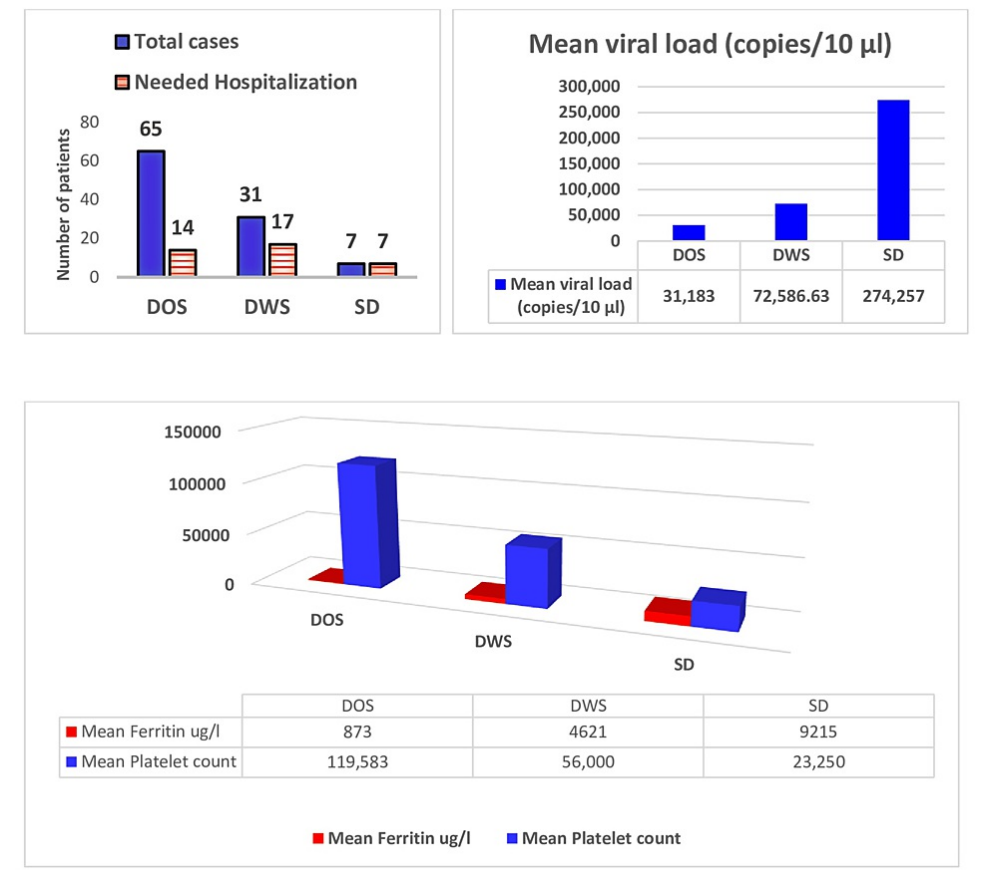
Correlation of dengue viral load, ferritin level, and platelet count with disease severity DOS: Dengue without warning signs, DWS: Dengue with warning signs, SD: Severe dengue

Among the 103 dengue patients, 38 (36.83%) required hospitalization. Calculating mean viral load, patients having SD had the highest mean viral load. The mean ferritin level was significantly high in SD and inversely related to platelet counts (Figure 7). An increase in hematocrit level >20% was seen in 25 patients; <20% increase was seen in 54 patients; and normal levels in 24 patients.

Correlation of dengue viral load with IgG reactive cases

**Figure 8:**
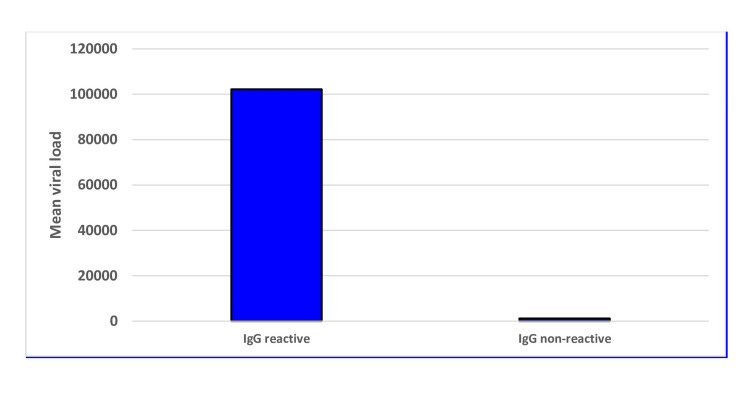
Correlation of dengue viral load with IgG reactive cases

In IgG reactive cases, the initial viral load was very high (mean value = 102195.47 copies/10µl) than in IgG non-reactive (mean value = 1195.38 copies/10µl) cases (Figure 8).

## Discussion

Result of dengue NS1 antigen

Among 103 NS1 reactive patients, the mean age was 29.4 (SD=17.4), and the data didn’t show any linear association with age for NS1 reactive patients (p = 0.829). Murhekar et al. found that most laboratory tests confirmed dengue patients were older than 20 years [13]. Moreover, the data of the study didn’t indicate any association for sex with NS1 reactivity. The incidence of NS1 reactive cases was highest in the post-monsoon season (72.8%) due to favorable conditions for mosquito breeding, followed by the monsoon (16.5%) and winter (10.6%) seasons. Dengue incidence was significantly associated with the number of rainy days in the preceding month [14,15]. To study the correlation of NS1 reactive cases with various laboratory parameters, we found a significant decrease in platelets as well as total leukocyte count (p<0.0001) in NS1 reactive patients than in NS1 non-reactive cases. This finding was the same for Ingale et al., Kanthikar et al., and Badave et al. [16-18]. The liver enzymes were significantly elevated as a result of reactive hepatitis or hepatocyte injury by dengue virus in NS1 reactive patients (p<0.0001) compared to the NS1 non-reactive cases. Kalluru et al. also found that the mean values of aspartate aminotransferase (AST) and alanine transaminase (ALT) are higher in SD than in DOS and suggested a correlation between increased transaminase levels and disease severity [19].

Presence of secondary infection deteriorates disease parameters

To find the effect of secondary infections, the data was classified according to the primary and secondary infections. We found around 56.31% of NS1 reactive individuals had secondary dengue infection, and the data of the laboratory parameters were interpreted accordingly among secondary and primary infected individuals. We have found a significant depletion (p = 0.0087) of platelet count, suggesting the worsening effect due to secondary infection. On the other hand, we have also found that SGOT, SGPT, and ALP levels were also significantly elevated for IgG reactive as well as secondary dengue-infected individuals. Swamy et al. also found a significant rise in liver enzymes in patients with secondary dengue infection [20]. However, we didn’t find any significant difference in terms of total leukocyte count. Antibody-dependent enhancement (ADE) in dengue, mediated by IgG, enhances viral entry into immune cells and promotes viral replication, leading to increased viral load and pro-inflammatory responses, which are the major underlying mechanisms of SD in secondary DENV infection.

Duration and severity of illness correlate with laboratory parameters and viral counts

As there was no mortality, duration, and severity of illness for NS1 reactive patients are considered clinical outcomes, and this study correlates the clinical outcome with the laboratory parameters and viral load. Monitoring the hematocrit level is important for fluid management in dengue cases. An increase in hematocrit value considering the peak value and its baseline level is an important marker for disease severity rather than the absolute value. There was a significant fall in platelet count in group C compared to group A (p = 0.0002) and group B (p = 0.01) but no significant change had been observed between group A and group B. Decreased platelet count may lead to SD. On the other hand, to assess the impact of the viral load of a patient on the duration of illness, we found a significantly higher viral load in group C compared to group A (p = 0.0037) and group B (p = 0.0032) but no significant difference was observed between group A and group B. In this study, the patients having SD had a high mean viral load and low platelet count, which was similar to other studies [21,22]. Again, high viral load correlates with thrombocytopenia. Nunes et al. and Vaughn et al. also found that higher viraemic titers were associated with increased disease severity. We found a strong correlation between increased ferritin levels and the severity of dengue infection. Dengue patients with elevated serum ferritin levels have to be monitored for the severe form of the disease, which leads to the activation of the immune system and abnormal coagulation [23]. In the case of liver enzymes, both SGOT and SGPT were significantly high in group C compared to group A and group B (p = 0.02) but no significant change had been observed between group A and group B.

## Conclusions

In this study, we tried to find out the correlation between viral factors and host components, which is reflected as laboratory parameters. Initial viral load, liver enzymes, and serum ferritin could predict the outcome of dengue. The higher the initial viral load, the longer the duration of illness. Moreover, we found a positive correlation between viral load with thrombocytopenia and secondary infection. The clinical progression of dengue NS1-reactive patients with IgG-positive patients has to be monitored carefully. Poor outcome of dengue infection in the form of increased duration of illness, disease severity, the need for hospital admission, and the presence or absence of any complication depends on concurrent associations between high serum ferritin, increased hematocrit percentage than baseline value, thrombocytopenia, secondary infection, an increase in liver enzymes, and increased initial viral load.
